# Three-Way Interaction Effect of Job Insecurity, Job Embeddedness and Career Stage on Life Satisfaction in A Digital Era

**DOI:** 10.3390/ijerph16091580

**Published:** 2019-05-06

**Authors:** Muhammad Rafiq, Tachia Chin

**Affiliations:** School of Management, Zhejiang University of Technology, Hangzhou 310023, China; rafiq109@yahoo.com

**Keywords:** job insecurity, life satisfaction, job embeddedness, career, China

## Abstract

Along with the severe global employment challenges caused by the rapid rise of digital technologies, the job insecurity (JI)–life satisfaction (LS) association has attracted increasing attention. However, there is still a dearth of studies investigating the crucial boundary conditions of JI–LS relationships in non-Western contexts. To fill this gap, we choose China, the world’s largest emerging economy, which is undergoing a radical digital transformation, as our research setting. Building on the conservation of resource (COR) theory, we focus on exploring two critical buffers of the JI–LS mechanism, of which job embeddedness (JE) characterizes a significant psychological resource and career stage embodies the time dynamics of this model. Data were collected from a sample of 317 Chinese media organization employees and were analyzed by the moderated hierarchical multiple regression approach. Our results show that JI is negatively related to LS and this relationship becomes stronger when employees have low JE (vs. high). Further, this two-way interaction is moderated by career stage; the impact of JI on LS is (1) stronger only for mid–late career stage employees who experienced low JE, and (2) weaker also only for mid–late career stage employees who experienced high JE. This study enriches the existing body of knowledge on the JI–LS model by highlighting the three-way interaction effect of JI, a critical psychological resource (i.e., JE), and time effect (i.e., career stage) on LS; it implies that older people with a certain amount of career experience and resource accumulation may perceive the effect of JI on LS differently than younger people.

## 1. Introduction

Global organizations as the primary providers of jobs and careers have not recovered from the sharp economic decline triggered by the 2008 financial crisis; they are facing severe employment challenges caused by radial technological innovation that encourages the use of smart machines and robots over human workers [1,2]. Evidence indicates that, apart from the less-skilled occupations, more and more middle-class jobs are becoming obsolete due to the high-speed advancement of science and technology [3,4]. Along with this new wave of unemployment across the globe, job insecurity and its impacts on people’s overall well-being have been drawing increasing attention [5,6,7]. 

Although quite a few studies have examined the detrimental effects of JI on life satisfaction (LS) and related psychological well-being outcomes [5,6,8,9,10], updated research is much needed for achieving a deeper, more comprehensive and systematic understanding of JI–LS relationships in today’s ever-changing workplace as some critical gaps remain. First, as indicated earlier, given the rising popularity of digitalization and automation is intensifying job losses in various occupations, JI will continue to have a significant and complex impact on most workers’ lives, which requires further investigation [10]. Second, while European scholars tend to analyze the influence of JI on employee health and well-being [5,8,11,12], prior research conducted in Asia, particularly in emerging markets, seems to focus more on employee outputs important to employers, such as work attitude and job performance [13,14]. It is thus imperative to link JI to worker well-being in such contexts. Third, to better manage the effects of JI, scholars have called for more research to identify and test potential moderators between JI and outcomes from a more pragmatic point of view [15]. However, there is limited evidence addressing the boundary conditions of JI–LS mechanisms. 

Considering the gaps illustrated above, this paper chooses China, the world’s largest emerging country with a high growth rate over recent decades, as our research setting. China, despite its rapid economic growth, has not been spared from the negative impacts of job insecurity; however, research efforts probing this area remain sparse. Already initial reports indicate a diverging trend between job insecurity elements in Western culture and Chinese culture. Harmony, stability, and safety, which are the cornerstones of Chinese culture, interestingly seem to exacerbate the ill-effects entailed by job insecurity. Certainly, more comprehensive empirical studies based on Chinese samples are warranted to elucidate the key underlying mechanisms pertaining to job insecurity. With extensive government support, this nation is undergoing fundamental changes towards digitalization, and as an emblematic transition economy in Asia, it is particularly suitable for our study to identify the key moderators of the JI–LS mechanisms during this transformation. 

According to the conservation of resources (COR) theory [16], people with more powerful psychological forces may be better equipped to cope with stressors emerging from adverse work conditions, as the perceived threat of losing valuable resources is relatively low. Scholars have revealed that, in turbulent times, embedded employees are less motivated to leave their jobs and feel more satisfied with their life because their abundant resources reduce their sense of anxiety [17,18]. Following this theoretical logic, we further argue that job embeddedness that reflects a collection of critical psychological resources [19] should be taken into consideration when identifying key moderators of JI–LS relations in an insecure or unstable work environment.

Considering time-related changes regarding psychological resources have not been clearly addressed in the COR or job embeddedness literature [17,20], we thus choose a career stage that reflects the time dynamics of an occupation and corresponding career-related resource changes [21] as another key moderator in our proposed model. This may help us gain a better understanding of the boundary conditions of the JI–LS mechanism at a critical juncture, namely a transitional phase towards digitalization. More specifically, we first predict a moderating effect of JE on the JI–LS relation, followed by a three-way interaction assumption in which career stage is posited to moderate the two-way interaction effect of JI and JE on LS.

This research makes three main contributions to the existing body of knowledge: First and foremost, we enrich the job insecurity literature by identifying two buffers (i.e., job embeddedness and career stage) of the JI–LS relation as well as their interacting impact on such associations. Second, our research to a certain extent adds new value to the COR theory by highlighting the importance of appropriately timing critical psychological resource inputs and how to cope with the changes of such resources during a specific transformational period of time. With the use of valuable first-hand data, we also offer abundant practical implications for media organizations in China and other non-Western contexts.

## 2. Literature Review and Hypotheses Development

Job security (JI) is widely defined as “the subjectively perceived and undesired possibility to lose the present job in the future, as well as the fear or worries related to this possibility of job loss” (p. 365 in [11]), while a large number of researchers have discovered that JI can be regarded as an indispensable job stressor in a variety of work stress models (e.g., [10,22,23,24]). Scholars also suggest that JI essentially functions as a typical work stressor, leading to job strain, emotional exhaustion, and work–life conflict [25]. In some serious cases, employees may suffer burnout so that they lose control over both their work and life, whereby their overall health and well-being are undermined. This is particularly true for those with a relatively low income; for them JI implies not only the threat of losing a job, but also the fear of losing valuable job-related resources, which usually has a heavy financial burden on their life. Echoing this point of view, since the 2008–2009 global financial crisis triggered fierce employment challenges, JI has become a prominent worldwide phenomenon and thereby has become one of the most-discussed topics in the health literature over the last decade [26]. As indicated earlier, more empirical studies investigating the impact of JI on overall well-being in non-Western contexts are needed. On the other hand, recently, Hu et al. [6] suggested that there are numerous studies already confirming the negative relationship between JI and LS, but rapidly changing working situations may make JI more salient to today’s employees. We thus hypothesize:

**Hypothesis 1** **(H1).** *Job insecurity will be negatively related to employees’ life satisfaction in China*.

### 2.1. Moderating Role of Job Embeddedness

As noted in the introduction, to further enhance our understanding of the relationship between JI and LS, there is a need to theoretically and empirically identify the boundary conditions for this relationship [10,27,28]. More specifically, it is vital to address the key buffering mechanisms of the JI–LS association. Van Dijk [29] stated that psychological resources are the foremost factor in individuals’ work/life that help them to cope with adverse circumstances. Körner et al. [30] suggested that rapid advancement in automation and digitalization in emerging and low-income countries continuously change contemporary working conditions that may negatively affect employees’ psychological conditions or resources; consequently, employee health has already become a matter of debate. Based on the conservation of resource (COR) perspective, Probst et al. [31] claimed that critical psychological resources have been shown to buffer the relationships between employees’ perceptions of JI and their behavior outcomes. However, while the importance of a variety of psychological resources has been articulated, limited research hitherto has examined the moderating role of psychological resources in the JI–LS relation. We, therefore, attempt to address the buffering impact of a critical psychological resource on the JI–LS relation here. 

Allen et al. [17] suggested that job embeddedness (JE) represents an important psychological resource that helps impair employees’ adverse feelings and emotions in a more efficient way. Mitchell et al. [19] state that JE is the collection of psychological factors that keep people in their present organizations that is defined by the notions of fit, links, and sacrifice, operating between employees and their organization and community. Fit is the extent to which one’s ability is compatible with the organization and community. Links reflect informal and formal connections that an individual has within the organization or community. Sacrifice is the perceived cost (i.e., material or psychological benefits) that an individual would incur if he/she decided to leave his/her current organization or residential community. Individuals who are highly embedded in their organizations and communities perceive themselves as a good fit, have a series of connections (i.e., links), and cognize significant personal sacrifices associated with leaving [15]. The construct may actually imply two sub-dimensions: Organizational (on the job) embeddedness and community (off the job) embeddedness. However, many past studies have regarded JE as a unidimensional construct and examined how it directly links to a wide range of individual-level outcomes (e.g., work attitudes and behavior) [17,32] because JE, as a broad perceptual construct, consists of social, financial, psychological, and environmental components that entangle people within a specific psychological web [33]. We follow their suggestions in this paper.

The COR theory by Hobfoll [16] posits that individuals are motivated to gain, protect, and build resources (e.g., objects, personal characteristics, conditions, or energies)—they feel threatened when speculating about the potential or experienced loss of valued resources [20]. JI presents such a threat, as it implies involuntary unemployment and the loss of job-associated resources [34]. We therefore assume that the relationship between JI and LS should be negative. It is worth noting that the COR theory also advocates the creation of a “resource caravan” (p. 6 in [23]) and encourages taking advantage of the resources at hand to cope with the fear of losing resources. It thus seems plausible to assume that the possession of greater resources may mitigate the negative JI–LS association. Following this logic, we argue that JE, as a powerful psychological resource in the conservation pool, is very likely to buffer the JI–LS relationship. We thus predict:

**Hypothesis 2** **(H2).** *Job embeddedness will moderate the negative relationship between job insecurity and life satisfaction, such that this negative relationship will be stronger when employees are less embedded than more embedded*.

### 2.2. The Moderating Role of Career Stage

As illustrated, the time dynamics of career-related resource changes may be a critical issue that has not been comprehensively addressed in the COR and JE literature [17,20]. With an attempt to fill this gap, we therefore include another moderator, career stage, in our model, whereby the boundary condition of the JI-LS relationship can be better characterized. 

Career stage theory has been used widely to describe several individuals’ work attitudes and behaviors across the span of their overall working life. According to this theory, individuals go through three career stages. Each of the three stages is associated with particular career concerns: Exploration, establishment (early career), maintenance (mid-career), and disengagement (late career) [35,36,37]. More specifically, at the early career stage, individuals are mostly concerned with identifying their interests and capabilities, and may frequently shift their attention and efforts to changing their professions or organizations. At the mid-career stage, individuals often focus on maintaining their current job status with a strong desire to achieve stability and constant growth in their careers; they may emphasize achievement, promotion, independence, and upward mobility. At the late career stage, individuals tend to psychologically detach themselves from their job, and their performance level is very likely to decline.

Shane and Heckhausen [38] further state that resources play an important role in an individual’s well-being at different career stages, because the career stage theory builds on the premise that what makes a gain or loss in an individual’s careers mainly relies on ontogenetic characteristics, changes, and conditions [39]. Following this logic, individuals at the mid–late career stage may become overcautious and thus be eager to avoid all kinds of resource loss. On the contrary, individuals at the early career stage are more likely to maximize resource gain than minimize resource losses [40], as they think they have plenty of time to seek new resources. It is evident that the distinctive set of work attitudes, intentions, and concerns of individuals may change according to their different career stages.

As far as the moderating role of career stage is concerned, previous literature has suggested that career stage can buffer quite a few relations between individual perceptions/feelings with attitudinal and behavioral outcomes; for example, turnover–turnover intent [21], JE–innovation-related behavior [41,42], and satisfaction–performance [43]. Referring to the theoretical logic demonstrated above, we further assume that the three-way interaction of JE, career stage and JI is very likely to exist. More specifically, while employees in the mid–late career stage are more concerned with career stability and job security [41], they are less likely to leave the company. As time passes by, they will get older but will have developed valuable connections and enjoyed accrued benefits, and thereby have become highly embedded in their organizations [41,44]. In contrast, nowadays employees in early career stages are less inclined to be highly embedded within their organizations because many of them have grown up in an era of low loyalty towards one employer and low expectations for long-term employment with one organization due to the increasing level of perceived JI. Research also shows that early career stage employees do not have a strong desire for job security nor do they pursue a high level of emotional attachment or job embeddedness with a company [45]. In general, people at the early career stage are more willing to take initiative when it comes to career matters.

Taking together the foregoing arguments, we further propose that career stage will moderate the two-way interaction effect of JI and JE on LS, such that the interaction effect will be (1) strongest for the mid–late career stage with low JE compared to early career stage employees and (2) weakest for the mid–late career stage with high JE compared to early career stage employees.

**Hypothesis 3a** **(H3a).** *Career stage moderates the two-way interaction effect of job insecurity and job embeddedness on life satisfaction, such that the negative relationship between job insecurity and life satisfaction is stronger for the mid–late career stage with lower job embeddedness compared to their early career stage counterparts*.

**Hypothesis 3b** **(H3b).** *Career stage moderates the two-way interaction effect of job insecurity and job embeddedness on life satisfaction, such that the negative relationship between job insecurity and life satisfaction is weaker for mid–late career stage with higher job embeddedness compared to their early career stage counterparts*.

## 3. Methods

### 3.1. Sample and Procedures

As addressed in the introduction, China is an appropriate setting for our research. While employees of this nation’s traditional media industry are suffering from intensifying unemployment pressure due to the increasing popularity of information technology, we thus chose three traditional media organizations in Beijing to conduct field surveys. Data were collected from March to June of 2018. We had a contact person who is a good friend of our team leader and has worked as an executive in one of the three sample firms; with sufficient background knowledge, he helped us avoid the extraneous influence when selecting the appropriate sample firms and facilitated our communication with the key persons of each company for arranging data collection.

Before the formal field surveys, the contact person first talked with the manager of the HR department of each organization and introduced the leader of our research team to them via telephone. Then, our team leader contacted the managers of the HR departments of all firms in person and requested a formal appointment to collect data. With their assistance, our research team visited the participating companies one by one to hand over to the HR managers our formal questionnaires. In each media organization, one of their HR officers was assigned to help us distribute and collect the questionnaires. To minimize common method bias [46], our survey questionnaire included a cover letter explaining the general aim of the study, followed by a clear guideline illustrating the sound protection of the privacy of participants. We also used a time-lagged design—the predictors (JI) and moderators (JE and career stage) were measured at Time 1, and the dependent variable (LS) at Time 2 (three months later).

At Time 1, 390 questionnaires were distributed to the respondents, from whom 369 usable sets of data were obtained. At Time 2, only 317 of the 369 respondents completed the survey, indicating an 85.90% response rate. Of the participating employees, 54% were female, the mean age was 38.35 years (SD = 1.21), 43% were married, and 66.89% reported having a bachelor’s degree. 

### 3.2. Measures

Job insecurity: We used a four-item scale developed by De Witte [47] to measure job insecurity (Time 1 questionnaire). Response options ranged from 1, “strongly disagree”, to 5, “strongly agree.” Sample items are “I feel insecure about the future of my job” and “I am sure I can keep my job’ (reverse coded)”. Cronbach’s alpha was 0.77.

Life satisfaction: We used Diener et al. [48] five-item scale to measure employees’ life satisfaction (Time 2 questionnaire). Sample items are “So far I have gotten the important things I want in life” and “In most ways, my life is close to my ideal”. Response options ranged from 1, “strongly disagree”, to 5, “strongly agree.” Cronbach’s alpha was 0.90.

Job embeddedness: We used Crossley et al. [49] seven-item scale to measure job embeddedness (Time 1 questionnaire). Sample items are “I am tightly connected to this organization” and “I feel tied to this organization”. Response options ranged from 1, “strongly disagree”, to 5, “strongly agree.” Cronbach’s alpha was 0.91.

Career stage: Career stage was operationalized using the convention designated by Gould and Hawkins [43], length of employees’ self-reported tenure in the organization (Time 1 questionnaire). Moreover, consistent with the prior studies [21,43,50], cut-off criterion was implied for career stages in this study as follows: Early career stage (n = 154; tenure ≤ two years) and mid–late career stage (n = 163; tenure > two years). Career stage was measured as a variable (1 = early career stage employees, 2 = mid–late career stage employees).

Control variables: Research suggests gender, age, marital status, and education relate to JI and LS [51]. Since we are interested in analyzing the association between JI, LS, and career stages, the demographic variables shown above were controlled for in data analyses to rule out alternative interpretations. These variables were measured as: Respondent’s gender (male = 1; female = 0), respondent’s status (married = 1; single or other = 0), age (in years), and education (bachelor = 1; master = 2; PhD = 3).

### 3.3. Analysis

Firstly, we used confirmatory factor analysis (CFA) to validate our multiple items scales [52]. Next, we used moderated multiple hierarchical regression analysis to test the three-way interaction effect. To test the significance of interaction, a simple slope examination was performed [53,54]. All analyses were performed in AMOS and SPSS.

## 4. Results

### 4.1. Confirmatory Factor Analysis

For a more rigorous psychometric property of the scales, CFA using AMOS-23 was employed here. Firstly, we conducted a separate CFA for all study variables (excluding career stage, which was a single-item scale). The model Chi-square (*χ*^2^), the ratio of *χ*^2^ to degrees of freedom (*χ*^2^/*df*), Tucker-Lewis index (TLI), comparative fit index (CFI), and root mean square error of approximation (RMSEA) were used to assess the overall model fit. To confirm a model fits the data well, Hu and Bentler [55] recommend that the value of *χ*^2^/*df* be less than 3, the cutoff value of TLI and CFI be 0.90 or above, and a good fit for RMSEA is less than 0.08.

Next, we estimated the full measurement model with all latent variables (JI, JE, and LS) in a single model. The CFA results indicated that our four-factor model (*χ*^2^ = 164.82; *df* = 71; CFI = 0.96; TLI = 0.95; RMSEA = 0.06; SRMR = 0.05) had a good fit to data. Additionally, CFA results showed that all observable indicators loaded significantly, being higher than 0.5, on their respective latent scales. As stated above, the Cronbach’s alpha scores for all study measures were above 0.77. Taken together, these results suggest the variables had acceptable internal validity. With regard to discriminant validity, we compared our restricted and unrestricted model with any lower-level factor structure by alternately constraining pairwise correlations between the latent factors at 1.0. All the lower-level factor models had significantly worse model fits, suggesting the factors were distinct.

Additionally, the variety of evidence surrounding discriminant validity may reflect the fact that the data used in the current study is typically cross-sectional and subject to common method bias that enhances the problem of establishing discriminant validity. Furthermore, we conducted a single-factor test to evaluate potential common method bias [46]. The test yields a poor fit (*χ*^2^ = 963.04; *df* = 77; CFI = 0.62; TLI = 0.56; RMSEA = 0.19; SRMR = 0.13), specifying that the indicators do not load on one general factor.

### 4.2. Descriptive and Correlation Analyses

Table 1 presents the means, standard deviations, and correlations for all study variables. The results shows that JI was negatively correlated with employees’ LS (*r* = –0.17, *p* < 0.01), JE (*r* = –0. 23, *p* < 0.01), and career stage (*r* = 0.14, *p* < 0.05). JE was positively related to employees’ LS (*r* = 0.58, *p* < 0.01), and negatively related to career stage (*r* = –0.27, *p* < 0.01). Meanwhile, career stage was significantly related to employees’ LS (*r* = –0.53, *p* < 0.01). These results provide a solid foundation for regression analysis.

### 4.3. Hypotheses Testing

We conducted a four-step moderated hierarchical multiple regression analysis to test our hypotheses. In the present study, four regression models were established for JI and LS. Step 1 controlled for sex, age, marital status and education. All three predictors were added to Step 2. Step 3 tested the moderating effect by adding two-way interaction. Finally, Step 4 examined the moderating effect of career stage by adding the three-way interaction. Before analysis, the predictors were mean-centered to reduce any multicollinearity [53].

All the results of the moderated hierarchical multiple regression analyses are presented in Table 2. Model 2 was used to test the main effects. Hypothesis 1 stated that JI was negatively associated with employees’ LS (*β* = −0.18, *p* < 0.05), after controlling for employees’ sex, age, marital status, and education.

**Figure 1:**
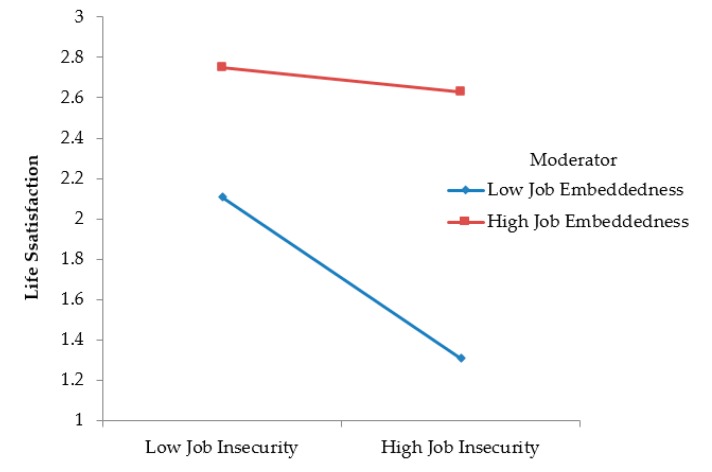
The two-way interaction effects of job insecurity and job embeddedness on employees’ life satisfaction.

Hypothesis 2 predicted that the relationship between JI and LS was moderated by JE, such that the relationship was stronger when JE was low (vs. high). As shown, Model 3 adds interaction into the model, which enables testing the hypothesis that involves the moderating effects of JE on the effect of JI on LS. The results in Model 3 show that the coefficient for the interaction terms of JI and JE was significant (*β* = 0.17, *p* < 0.05). We plotted Figure 1 to illustrate the simple slope difference regarding the effect of JI under high or low (±1 SD) levels of JE. Figure 1 clearly displays that when JE is low, JI has a stronger negative effect on LS. Additionally, subgroup analysis reveals that respondents who were below the mean on JE reported a correlation of –0.53 (*N* = 137) between JI and LS. In contrast, respondents who were above the mean on JE reported a correlation of –0.35 (*N* = 180) for the relationship between JI and LS. These two effect sizes were significantly different. Thus, Hypothesis 2, which proposed that the relationship between JI and LS is more moderated by JE for less embedded employees, was supported.

**Figure 2:**
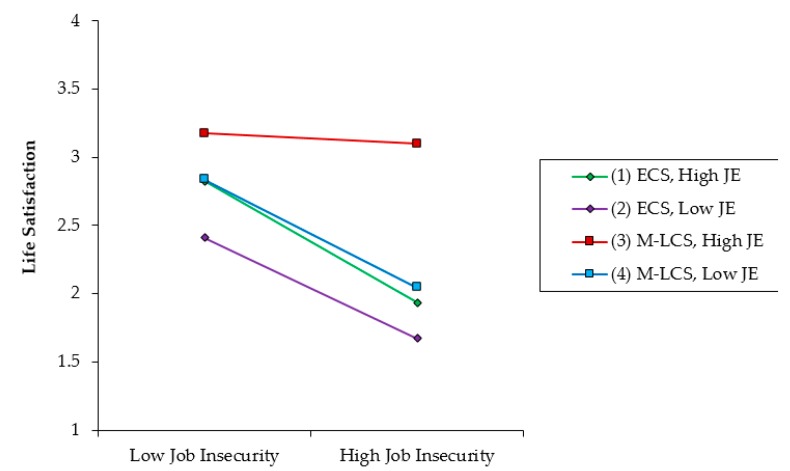
The three-way interaction effects of job insecurity, job embeddedness, and career stage on life satisfaction. Note: JE = job embeddedness; ECS = early career stage; M–LCS = mid–late career stage.

Hypothesis 3 predicted that the relationship between JI and LS would be (a) stronger for individuals who have low JE at mid–late career stage than for early career stages and (b) weaker for individuals who have high JE at the mid–late career stage than early career stages. Model 4 adds the three-way interaction involving JI, JE, and career stages into the regression analysis. The three-way interaction’s coefficient on LS is significant (*β* = –0.43, *p* < 0.05). To more formally test the differences across the four lines displayed in Figure 2, we used Dawson and Richter’s [54] recommended approach for comparing slopes. Using this method, we observed that the slopes for the group of mid–late career stage employees with both (i.e., low and high) JE for JI was significantly different from the early career stage group (*p* > 0.05). Furthermore, Figure 2 clearly shows that the relationship between JI and LS is a) stronger for the mid–late career stage with low JE than for the early career stage and b) weaker for the mid-late career stage with high job embeddedness than for the early career stage. Thus, Hypotheses 3 was supported. 

## 5. Discussion

Overall, our four hypotheses have been fully examined. Our findings show that JI is negatively related to LS. Furthermore, JE acts as a moderator of the JI–LS association, in such a way that the JI–LS relation is stronger among low-embedded employees as compared to high-embedded ones. We have also discovered that that career stage moderates this two-way interaction of JI and JE on LS. Our results illustrate that this three-way interaction is only significant for the mid–late career stage, such that this negative relationship between JI and LS is (1) strongest for the mid–late career stage with low JE and (2) weakest for the mid-late career stage with high JE. 

There are several ways in which these findings contribute to the literature. Firstly, the results of this study show a negative relationship between JI and LS during a transformational period towards digitalization in China, providing potent support to the previous research that demonstrated the negative association between JI and subjective well-being in the same context [10]. Whereas quite a few Western scholars are calling for more attention being paid to the deleterious impact of JI on people’s health [22,27], our paper partly responds to their appeal by showing that JI not only elicits work-related and occupational stress, but also undermines overall well-being in different cultural and industrial settings. 

Second, our results examine the proposed two-way interaction whereby JE moderates the JI–LS relationship, thus delivering a deeper understanding of how a critical psychological resource like JE buffers the JI–LS relationship. According to the COR theory, individuals with fewer resources are more vulnerable to further future loss and thereby are eager to protect their resources in the face of anxiety and fear [56]. In other words, when individuals have abundant resources, their satisfaction towards life may not decline immediately or quickly, even when facing a high level of JI. This is because, for such resourceful people, it usually takes far longer than for those with limited resources to perceive harms of job loss. Our finding that JE, as a crucial psychological resource, indeed buffers the adverse effect of JI on LS offers precious empirical evidence to support the foregoing arguments, whereby we enrich the JE theory by adopting the COR perspective to identify the moderating effect of JE.

Our final and foremost imperative is to respond to scholars’ appeal for addressing the time dynamics of the COR theory [17,20,57]. This paper thus contributes to the existing body of knowledge by exploring the time effect of careers on the JI–LS mechanism, as the three-way interaction effect (i.e., JI, JE, and career stage) was found to be significant only for mid–late career stage employees. In other words, compared to young and early stage individuals, who in general have not accumulated a certain amount of resources, the life satisfaction of aged individuals at the mid–late career stage seems to be largely determined by their possession of critical psychological resources. More specifically, if such people have high job embeddedness, their sense of job insecurity and other related organizational stressors may have little impact on their subjective satisfaction in life as their risk tolerance is high under these circumstances. In contrast, if their level of job embeddedness is low, their JI may reversely become a critical detriment to their subjective well-being. As indicated by Thomas [40], the mid–late career stage is a very critical phase in people’s life span because individuals at this stage may become overcautious and sensitive about resource gains and losses.

We also provide valuable practical insights for enterprises and policy-makers, particularly in the field of occupational health care because automation and digitalization have largely reduced job vacancies, thereby placing increased pressure on workers. Our results suggest that only employees in the mid–late career stage are likely to show greater LS as an outcome of boosted JE. It implies that although organizations can help employees cope with job-related stress by boosting critical psychological resources like JE, managers should take employees’ career stages into account when attempting to reduce workers’ job insecurity by enhancing their embeddedness. In particular, organizations may need to re-think their policy in relation to employees’ well-being and, more specifically, pay closer attention to the needs of employees at different career stages. More explicitly, our research highlights that, in management practice, employees at different career stages may consider embeddedness and insecurity differently. So, today organizations need to adopt more tailored approaches to maximize their positive benefits; e.g., organizations need to wisely redress employee well-being-related initiatives that exclusively focus on the needs of early and mid–late career stage employees. From this sense, we further argue that the joint interaction of employees’ career stages and levels of job embeddedness may pose new challenges for global managers in allocating work to employees. 

### Limitations and Future Research

We acknowledge that this research is still subject to some limitations. First, this study not only focuses on a relatively small sample size but also relies on a single industry, which might limit the generalizability of the results. Future research is encouraged to include a more diverse and large sample of respondents, and to conduct studies in multiple contexts, industries, and countries. Second, the study relies solely on self-reported data, which could give rise to common method bias (CMB) [46]. While we adopted the time-lagged approach to control for sampling and employed Harman’s single factor analysis to mitigate CMB, we believe that CMB will not impair the methodological rigor of this paper. However, despite the precautions mentioned above, we are still unable to completely ignore the possibility of CMB. As such, it is recommended future studies use a greater variety of methods; for example, mixing qualitative and quantitative methods. Third, in this study, we chose the globally recognized, yet relatively short versions of JI and JE scales. However, in view of the contextual complexity, scholars have suggested employing more comprehensive JI and JE measures [58,59]. It is thus beneficial for future research to adopt different JI and JE scales to investigate relevant issues. Fourth, this study focuses on only two career stages (mid–late and early career stage). We also recommend that future research should include three career stages (early, mid, and late career stage) for better understanding. Finally, considering the three-way interaction analysis is exploratory in nature, more possible relationships among the three variables may be found. Hence, we also suggest that the joint interaction of employees’ career stages and levels of job embeddedness could pose new challenges for global managers in allocating work to employees. For instance, early-stage employees may bear the difficulty of tasks independently and will not feel unhappy if they fail and may choose to leave the organization without hesitation. Oppositely, mid–late career stage employees may shun risky job assignments and expect more support from their colleagues, as they in general are more emotionally attached to the organization. 

## 6. Conclusions

For organizations, digitalization offers transformative benefits. At the same time, for employees, these same tools often bring a sense of JI. Organizations are continuously trying to use many strategies to manage JI and its consequences for the workforce. To help begin anew, this research set out to explore the three-way effect of JI, JE (i.e., critical psychological resources), and career stage (i.e., time effect) on LS in the fastest digitally-transforming economy, China. While this research is seen as exploratory research, it is clear that examining the JI–LS relationship in terms of psychological resources and time perspectives can produce valuable insights from both a theoretical and practical viewpoint. It is clear that organizations need to adopt a more tailored approach to managing JI-related consequences and have the courage to consider new ways to approach the issues and complexities of modern day living for their employees, targeting specific groups with relevant, tailor-made JI–LS-related initiatives. What has emerged from this study is the lack of attention being paid to younger workers and their JI–LS concerns, and this group in particular is therefore deserving of renewed consideration.

## Figures and Tables

**Table 1:** Mean, standard deviation, and bivariate correlation of research variables.

	1	2	3	4	5	6	7	8
1. Life satisfaction	1							
2. Job embeddedness	0.58 **	1						
3. Career stage	−0.53 **	−0.27 **	1					
4. Job insecurity	−0.17 **	−0.23 **	0.14 *	1				
5. Sex	−0.03	−0.01	−0.02	0.04	1			
6. Age	−0.12 *	−0.01	0.02	−0.03	0.01	1		
7. Marital status	0.04	0.03	0.01	0.02	−0.08	0.60 **	1	
8. Education	−0.05	0.04	0.06	0.02	0.09	0.19 **	0.15 **	1

Note: Significance at: ** *p* < 0.01 and * *p* < 0.05; n = 317.

**Table 2:** Results for moderated multiple hierarchical regression analyses for life satisfaction.

Predictor	Model 1	Model 2	Model 3	Model 4
	β	β	β	β
Control				
Sex	−0.02	−0.04	−0.04	−0.05
Marital status	0.40 **	0.41 **	0.38 **	0.34 **
Age	−0.21 **	−0.21 **	−0.19 **	−0.18 **
Education	−0.03	−0.03	−0.01	−0.02
Main effects				
JI		−0.18 *	−0.23 **	−0.28 **
JE		0.48 **	0.49 **	0.52 **
CS		−0.55 **	−0.57 **	−0.58 **
Two-way interactions				
JI × JE			0.17 *	0.14 *
JI × CS			−0.12	−0.19 *
JE × CS			−0.48 **	−0.36 **
Three-way interactions				
JI × JE × CS				−0.43 *
R2	0.04	0.21	0.28	0..31
R2 change		0.17 **	0.11 **	0.20 **

Note: Significance at: ** *p* < 0.01 and * *p* < 0.05; n = 317; JI = job insecurity; JE = job embeddedness; CS = career stage; unstandardized coefficients are shown.
